# De novo metastatic breast cancer in men vs women: a Swedish population-based cohort study

**DOI:** 10.1093/jncics/pkad050

**Published:** 2023-07-25

**Authors:** Aglaia Schiza, Irma Fredriksson, Malin Sund, Antonios Valachis

**Affiliations:** Science for Life Laboratory, Department of Immunology, Genetics and Pathology, Uppsala University, Uppsala, Sweden; Department of Oncology, Uppsala University Hospital, Uppsala, Sweden; Department of Molecular Medicine and Surgery, Karolinska Institutet, Stockholm, Sweden; Department of Breast, Endocrine Tumours and Sarcoma, Karolinska Comprehensive Cancer Center, Karolinska University Hospital, Stockholm, Sweden; Department of Surgical and Perioperative Sciences, Umeå University, Umeå, Sweden; Department of Surgery, University of Helsinki and Helsinki University Hospital, Helsinki, Finland; Science for Life Laboratory, Department of Immunology, Genetics and Pathology, Uppsala University, Uppsala, Sweden; Department of Oncology, Faculty of Medicine and Health, Orebro University, Orebro, Sweden

## Abstract

Current evidence on de novo metastatic breast cancer is based on data from women. This Swedish population-based cohort study compared the incidence over time and prognosis of de novo metastatic breast cancer between sexes using data from the Swedish National Quality Register for Breast Cancer. Joinpoint regression analysis was used to compare incidence trends in all stages (104 733 women, 648 men) and multivariate Cox regression analysis to investigate potential sex disparities in de novo metastatic breast cancer prognosis (6005 women, 41 men). For both sexes, increased trends were evident for cancer stages I and II, with a stabilizing trend at the later years for women, while stage III incidence remained stable. An increased trend for de novo metastatic breast cancer in women, and to a lesser extent in men, was observed. No difference in de novo metastatic breast cancer overall survival between sexes was observed (hazard ratio = 1.24; 95% confidence interval = 0.85 to 1.81). The comparable features in terms of incidence and prognosis of de novo metastatic breast cancer between sexes imply similarities, supporting the adoption of common treatment strategies.

De novo metastatic breast cancer refers to distant metastasis at the initial diagnosis, representing 5% of new breast cancer cases in high-income countries (1). Interestingly, the incidence of de novo metastatic breast cancer in women seems to be stable over time, unlike the increased incidence of early breast cancer (2,3). Moreover, patients with de novo metastatic breast cancer have a better prognosis than patients with recurrent metastatic breast cancer, supporting the notion that de novo metastatic breast cancer represents a separate clinical entity (4). The current evidence on de novo metastatic breast cancer is based on data only from women. Breast cancer in men is a rare, understudied disease, representing less than 1% of all breast cancer cases. Although the incidence of breast cancer in men seems to follow a similar increased trend as in women, there are substantial differences in patient- and tumor-related characteristics, with more advanced disease at diagnosis and overrepresentation of luminal breast cancer in men (5). No evidence exists, however, regarding de novo metastatic breast cancer incidence over time in men or their prognosis compared with women. We aimed to investigate the incidence of de novo metastatic breast cancer in both sexes within the same nationwide, population-based cohort and analyze potential sex-based disparities in terms of de novo metastatic breast cancer prognosis.

In this Swedish population-based cohort study, we identified all patients with invasive breast cancer, regardless of sex and stage, between 2008 and 2020 in the National Quality Register for Breast Cancer (NKBC). The clinical stage was determined according to the American Joint Committee on Cancer 7th edition staging manual (6). Because the NKBC has data only about tumor characteristics and treatment (7), we used the registry-based Breast Cancer Data Base Sweden 3.0 mega-linkage cohort based on linked data from the NKBC with other national registries to get access to unavailable information through the NKBC. Approval was granted by the Regional Ethics Committee, Stockholm (approval No. 2019-02610). Patients were categorized as having been diagnosed with de novo metastatic breast cancer if distant metastasis was evident within 3 months from the diagnosis date. For the incidence analysis, the whole cohort was used, with stage and sex as stratification factors. For the prognostic analysis, only patients with de novo metastatic breast cancer were included. For analysis of incidence trends over time, joinpoint regression models were fitted based on the logarithm of incidence rate per year (calculated as incidence per 100 000 inhabitants and standardized by the Swedish population in 2010) from 2008 to 2020. The best fitting log linear regression model was selected to identify the joinpoints (calendar year at diagnosis) when annual percentage changes differed statistically significantly (Joinpoint regression software [National Cancer Institute, Bethesda, MD]). Multivariable Cox regression analysis was used to investigate potential sex disparities in overall survival when adjusted for well-established breast cancer prognostic factors. Kaplan-Meier curves were used for visualization of overall survival between sexes (using SPSS, version 21, statistical software, IBM, Armonk, NY).

In total, 104 733 women and 648 men with breast cancer were included in the study cohort. The joinpoint regression analysis in women with breast cancer showed a statistically significant increased trend over time for stage I and II disease during the first time period (annual percentage change = 8.96 and 6.00, respectively), with a more stabilized trend at the later time points. A similar trend was observed for de novo metastatic breast cancer (annual percentage change = 2.58 during 2008-2017), whereas the incidence trend for stage III diseases remained stable (Figure 1, A).

**Figure 1.**
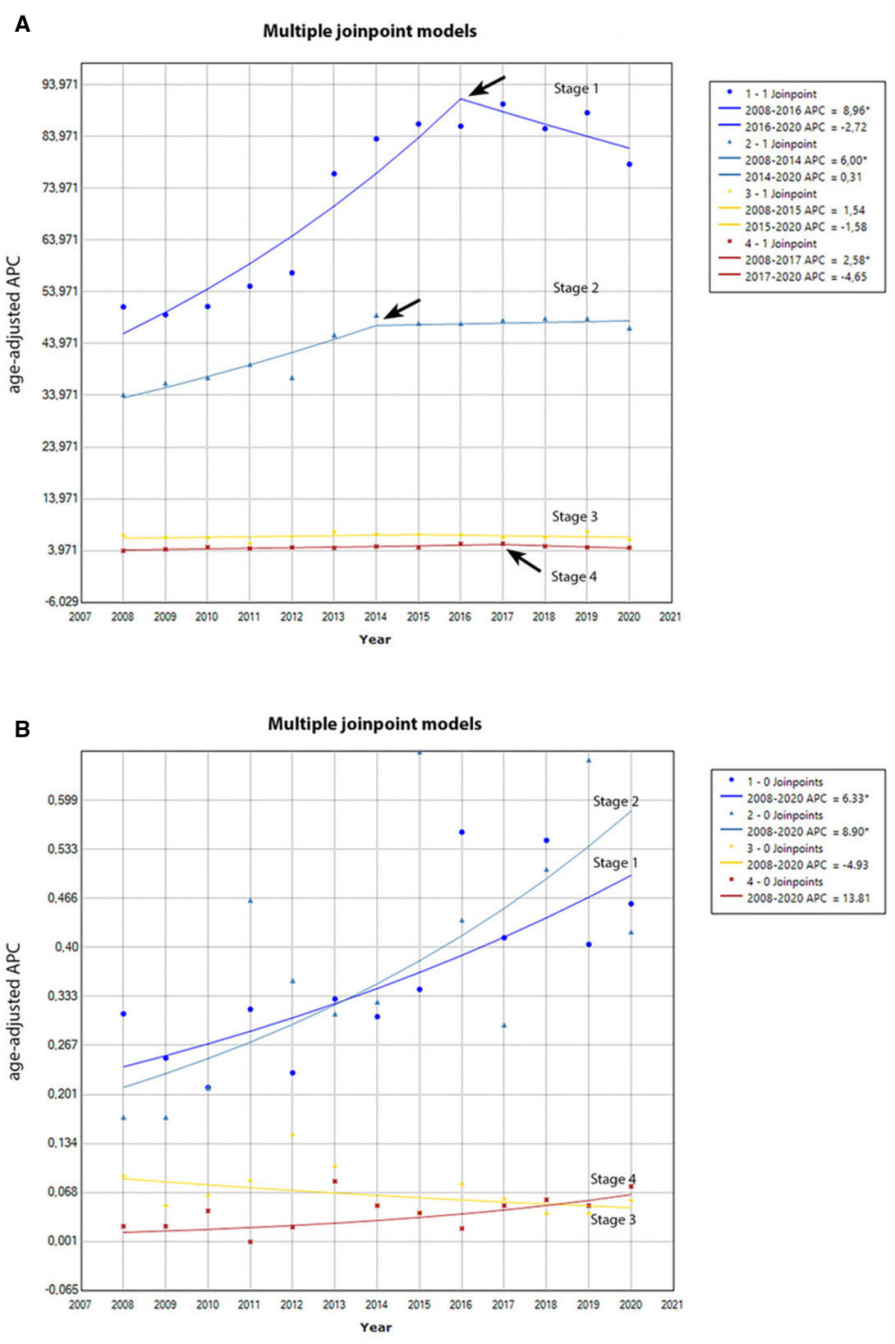
**A**) Stage-specific, age-adjusted incidence rate per 100 000 women with breast cancer, as determined using joinpoint regression, expressed by annual percent change (APC) between 2008 and 2020. APC is the percentage change in the incidence every year. APC* = the difference is statistically significant. A statistically significant increased trend over time for stage I and II disease as well as for stage IV disease during the first time period was observed (**black arrows**). **B**) Stage-specific, age-adjusted incidence rate per 100 000 men with breast cancer, as determined using joinpoint regression, expressed by APC between 2008 and 2020. APC is the percentage change in the incidence every year. APC* = the difference is statistically significant.

In men, an increased trend over time for stage I to II disease was evident (annual percentage change = 6.33 and 8.90, respectively), without a trend for stabilization. A similar increased trend, though nonstatistically significant, was observed for de novo metastatic breast cancer (annual percentage change = 13.81), whereas the incidence trend over time was stable for stage III disease (Figure 1, B).

For this analysis, 6005 women (99.3%) and 41 men (0.7%) with de novo metastatic breast cancer were included. Table 1 describes the demographics of eligible patients, where a statistically significant difference in terms of age at diagnosis was observed (median age = 70 years [range = 26-96 years] for women; mean age = 62 years [range = 47-83 years] for men). No difference regarding overall survival was observed between sexes in patients with de novo metastatic breast cancer (hazard ratio = 1.24; 95% confidence interval = 0.85 to 1.81) when adjusted for age, region, household income, breast cancer subtype, and surgery (Supplementary Figure 1, available online). The 5-year overall survival rates were 28% (95% confidence interval = 21% to 36%) and 25% (95% confidence interval = 18% to 33%) for women and men with de novo metastatic breast cancer, respectively.

**Table 1.** Summary of demographic (both self-reported and investigator classified) and clinical variables for female and male patients with de novo metastatic breast cancer^a^^,^^b^

Characteristic	Female (n = 6005)	Male (n = 41)	*P* ^c^
Age, median (range), y	70 (26-96)	62 (47-83)	.030
Household income, No. (%)	n = 5948	n = 41	.120
Q4	3282 (55.2)	16 (39)	
Q3	1325 (22.3)	12 (29.3)	
Q2	809 (13.6)	6 (14.6)	
Q1	532 (8.9)	7 (17.1)	
Region, No. (%)	n = 5961	n = 41	.748
Northern	714 (12)	6 (14.6)	
Stockholm-Gotland	889 (14.9)	6 (14.6)	
Uppsala-Örebro	1153 (19.3)	11 (26.8)	
South	1478 (24.8)	10 (24.4)	
Southeast	625 (10.5)	3 (7.3)	
Western (Halland)	1102 (18.5)	5 (12.2)	
Clinical T stage, No. (%)	n = 6005	n = 41	.739
T 0 to 1	1661 (27.7)	12 (29.3)	
T 2 to 4	4039 (67.3)	28 (68.3)	
Missing data	305 (5)	1 (0.4)	
Clinical N stage, No. (%)	n = 6005	n = 41	.179
cN+	2470 (41.1)	11 (26.8)	
cN‒	2950 (49.1)	25 (61)	
Missing data	585 (9.8)	5 (12.2)	
Estrogen receptor status, No. (%)			
Positive	3037 (50.6)	26 (63.4)	.232
Negative	615 (10.2)	4 (9.8)	
Missing data	2353 (39.2)	11 (26.8)	
Progesterone receptor status, No. (%)			
Positive	2218 (37.0)	20 (48.8)	.959
Negative	1059 (17.6)	7 (17.1)	
Missing data	2728 (45.4)	14 (34.1)	
Subtype according to immunohistochemistry, No. (%)	n = 6005	n = 41	.101
Luminal	2510 (41.8)	23 (56.1)	
HER2 positive	522 (8.7)	0 (0.0)	
Triple-negative breast cancer^d^	343 (5.7)	3 (7.3)	
Missing data	2630 (43.8)	15 (36.6)	
Breast surgery, No. (%)	n = 6005	n = 41	.774
Yes	240 (4)	2 (4.9)	
No	5765 (96)	39 (95.1)	
Axillary surgery, No. (%)	n = 6005	n = 41	.914
Yes	163 (2.7)	1 (2.4)	
No	5842 (97.3)	40 (97.6)	

a NKBC 2008-2020, BCBaSe 3.0, 2008 to 2019. NKBC = National Quality Register for Breast Cancer; BCBaSe 3.0 is a registry-based mega-linkage cohort based on linked data from NKBC with other National Registries; Q = quartile.

b Data on grade are not reported because the vast majority of the patients did not undergo surgery. For the few patients who did undergo surgery, the procedure was for the primary tumor and regional lymph node metastases.

c All *P* values reported were 2-sided; *P *< .05 was considered statistically significant.

d According to the Swedish guidelines, triple-negative breast cancer was defined as breast cancer that is estrogen receptor negative (ER < 10%), progesterone receptor negative (PgR < 10%), and HER2 negative.

This nationwide, population-based study offers new insights into the incidence trends over time in women and men with breast cancer, stratified by clinical stage, with special interest in de novo metastatic breast cancer; it also presents novel information about de novo metastatic breast cancer prognosis in men compared with women. Regarding incidence trends over time, 3 specific patterns deserve more attention. First, in line with previous studies from other countries, an increased incidence trend for early-stage breast cancer was observed in both sexes (8,9). Considering the stable incidence for stage III disease in men and women, the increased trend in earlier stages may indicate an increased breast cancer awareness for both sexes (10). Of note, in 2013 the clinical stage definition was changed to include a radiologic tumor size estimation, resulting in a potential stage migration toward higher disease stage over time. Second, a swift towards a more stable incidence trend for early-stage breast cancer in women appears during the later time period. Whether this incidence is a true effect that may be associated with changes in mammography screening behavior, a decline resulting from reduced use of menopausal hormone therapy (11,12), or a temporal effect is yet to be determined through further research in an expanded time period. Third, a potential increased incidence trend for de novo metastatic breast cancer mainly in women but also in men is suggested that contradicts findings from previous studies from other continents (3,13). Given the limited number of patients, we hypothesize that this trend may reflect the changing treatment landscape—namely, increasing use of neoadjuvant therapy, where staging procedures with imaging techniques are common practice.

Interestingly, no sex-related differences in de novo metastatic breast cancer prognosis were observed. Although we lack information about treatment strategies after de novo metastatic breast cancer diagnosis, our results imply that men may have access to new treatment strategies at the same level as women, thus gaining similar survival benefit. In addition, our results strengthen the current recommendations on treating men with similar principles as women with metastatic breast cancer.

Despite the inherent limitations associated with the retrospective nature of this study, our results offer some new insights into the clinical importance of de novo metastatic breast cancer in men—a rare entity. As the incidence trends and prognosis of de novo metastatic breast cancer between women and men seem to be comparable, supporting the hypothesis that this clinical entity shares similar features between sexes, the treatment strategies and adoption of new therapies for these patients should also be similar.

## Supplementary Material

pkad050_Supplementary_Data

## Data Availability

The data underlying this article cannot be shared due to restrictions by Swedish and European law to protect patient privacy. Data are available from register holders (Statistics Sweden, Swedish National Board of Health and Welfare, the Regional Cancer Center Stockholm Gotland) for researchers with relevant ethical approvals and who meet the criteria for access to confidential data.

## References

[1] Daily K , Douglas E, Romitti PA, Thomas A. Epidemiology of de novo metastatic breast cancer. Clin Breast Cancer. 2021;21(4):302-308. doi:10.1016/j.clbc.2021.01.017. 33750642

[2] Lei S , Zheng R, Zhang S, et al Global patterns of breast cancer incidence and mortality: a population-based cancer registry data analysis from 2000 to 2020. Cancer Commun (Lond). 2021;41(11):1183-1194. doi:10.1002/cac2.12207. 34399040 PMC8626596

[3] Malmgren JA , Mayer M, Atwood MK, Kaplan HG. Differential presentation and survival of de novo and recurrent metastatic breast cancer over time: 1990-2010. Breast Cancer Res Treat. 2018;167(2):579-590. doi:10.1007/s10549-017-4529-5. 29039120 PMC5790843

[4] den Brok WD , Speers CH, Gondara L, Baxter E, Tyldesley SK, Lohrisch CA. Survival with metastatic breast cancer based on initial presentation, de novo versus relapsed. Breast Cancer Res Treat. 2017;161(3):549-556. doi:10.1007/s10549-016-4080-9. 28000014

[5] Cardoso F , Bartlett JMS, Slaets L, et al Characterization of male breast cancer: results of the EORTC 10085/TBCRC/BIG/NABCG International Male Breast Cancer Program. Ann Oncol. 2018;29(2):405-417. doi:10.1093/annonc/mdx651. 29092024 PMC5834077

[6] Edge SB , Compton CC. The American Joint Committee on Cancer: the 7th edition of the AJCC cancer staging manual and the future of TNM. Ann Surg Oncol. 2010;17(6):1471-1474. doi:10.1245/s10434-010-0985-4. 20180029

[7] Lofgren L , Eloranta S, Krawiec K, Asterkvist A, Lönnqvist C, Sandelin K; steering group of the National Register for Breast Cancer. Validation of data quality in the Swedish National Register for Breast Cancer. BMC Public Health. 2019;19(1):495. doi:10.1186/s12889-019-6846-6. 31046737 PMC6498669

[8] Anderson WF , Jatoi I, Tse J, Rosenberg PS. Male breast cancer: a population-based comparison with female breast cancer. J Clin Oncol. 2010;28(2):232-239. doi:10.1200/JCO.2009.23.8162. 19996029 PMC2815713

[9] Konduri S , Singh M, Bobustuc G, Rovin R, Kassam A. Epidemiology of male breast cancer. Breast. 2020;54:8-14. doi:10.1016/j.breast.2020.08.010. 32866903 PMC7476060

[10] Nishimura Y , Acoba JD. Impact of breast cancer awareness month on public interest in the United States between 2012 and 2021: a Google trends analysis. Cancers (Basel). 2022;14(10):2534. doi:10.3390/cancers14102534. 35626141 PMC9140129

[11] Lagerlund M , Åkesson A, Zackrisson S. Population-based mammography screening attendance in Sweden 2017-2018: a cross-sectional register study to assess the impact of sociodemographic factors. Breast. 2021;59:16-26. doi:10.1016/j.breast.2021.05.011. 34118780 PMC8207312

[12] Zbuk K , Anand SS. Declining incidence of breast cancer after decreased use of hormone-replacement therapy: magnitude and time lags in different countries. J Epidemiol Community Health. 2012;66(1):1-7. doi:10.1136/jech.2008.083774. 21875869

[13] Bhoo-Pathy N , Verkooijen HM, Tan EY, et al Trends in presentation, management and survival of patients with de novo metastatic breast cancer in a Southeast Asian setting. Sci Rep. 2015;5:16252. doi:10.1038/srep16252. 26536962 PMC4633674

