# Supplementary material for: De novo metastatic breast cancer in men vs women: a Swedish population-based cohort study
Source: JNCI Cancer Spectr. 2023 Jul 25;7(4):pkad050. doi: 10.1093/jncics/pkad050 (PMC10444027; doi:10.1093/jncics/pkad050)
Supplement: pkad050_Supplementary_Data [file pkad050_supplementary_data.pdf]

Supplementary material

Supplementary Figure 1

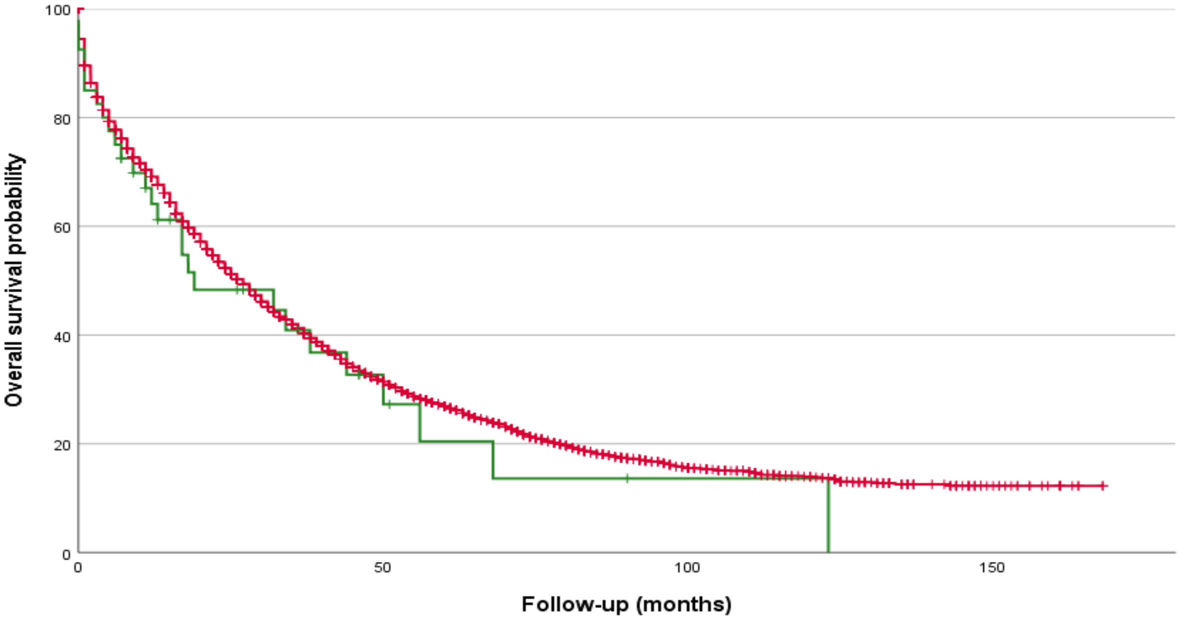

<u>Supplementary Figure 1</u>: *Kaplan-Meier survival curve for overall survival in the dnMBC analysis. This figure analyzes the survival of 6005 women and 41 men with dnMBC.*
